# Arrhythmias in Patients With Valvular Heart Disease: Gaps in Knowledge and the Way Forward

**DOI:** 10.3389/fcvm.2022.792559

**Published:** 2022-02-15

**Authors:** Maciej Kubala, Christian de Chillou, Yohann Bohbot, Patrizio Lancellotti, Maurice Enriquez-Sarano, Christophe Tribouilloy

**Affiliations:** ^1^Department of Cardiology, Amiens University Hospital, Amiens, France; ^2^Jules Verne University of Picardie, Amiens, France; ^3^Department of Cardiology, University Hospital Nancy, Vandœuvre lès Nancy, France; ^4^Department of Cardiology, GIGA Cardiovascular Sciences, University of Liège Hospital, Valvular Disease Clinic, CHU Sart Tilman, Liège, Belgium; ^5^Division of Cardiovascular Diseases and Internal Medicine, Mayo Clinic, Rochester, MN, United States

**Keywords:** valvular heart disease, atrial arrhythmia, ventricular arrhythmia, arrhythmic mitral valve prolapse, aortic stenosis, postoperative atrial fibrillation

## Abstract

The prevalence of both organic valvular heart disease (VHD) and cardiac arrhythmias is high in the general population, and their coexistence is common. Both VHD and arrhythmias in the elderly lead to an elevated risk of hospitalization and use of health services. However, the relationships of the two conditions is not fully understood and our understanding of their coexistence in terms of contemporary management and prognosis is still limited. VHD-induced left ventricular dysfunction/hypertrophy and left atrial dilation lead to both atrial and ventricular arrhythmias. On the other hand, arrhythmias can be considered as an independent condition resulting from a coexisting ischemic or non-ischemic substrate or idiopathic ectopy. Both atrial and ventricular VHD-induced arrhythmias may contribute to clinical worsening and be a turning point in the natural history of VHD. Symptoms developed in patients with VHD are not specific and may be attributable to hemodynamical consequences of valve disease but also to other cardiac conditions including arrhythmias which are notably prevalent in this population. The issue how to distinguish symptoms related to VHD from those related to atrial fibrillation (AF) during decision making process remains challenging. Moreover, AF is a traditional limit of echocardiography and an important source of errors in assessment of the severity of VHD. Despite recent progress in understanding the pathophysiology and prognosis of postoperative AF, many questions remain regarding its prevention and management. Furthermore, life-threatening ventricular arrhythmias can predispose patients with VHD to sudden cardiac death. Evidence for a putative link between arrhythmias and outcome in VHD is growing but available data on targeted therapies for VHD-related arrhythmias, including monitoring and catheter ablation, is scarce. Despite growing evidences, more research focused on the prognosis and optimal management of VHD-related arrhythmias is still required. We aimed to review the current evidence and identify gaps in knowledge about the prevalence, prognostic considerations, and treatment of atrial and ventricular arrhythmias in common subtypes of organic VHD.

## Introduction

Valvular heart diseases (VHD) are frequent in the population, with estimated prevalence of 2.5% increasing with age in relation with predominance of degenerative causes in the elderly (1, 2). Atrial fibrillation (AF) also is identified in 1-2% and premature ventricular contractions (PVC) in about 50% of the general population and their prevalence increase with age (3–5). While advancing age is common to VHD and arrhythmias, the character of this relation remains elusive, the appropriate detection and treatment of arrhythmias in patients with VHD and the impact of arrhythmias on the management of VHD remain poorly defined. The classical teaching is that in patients with VHD, atrial and ventricular arrhythmias (VAs) are harbingers of poor clinical outcome. However, most available data and clinical trials are focused on non-valvular arrhythmias. As a case in point mitral regurgitation (MR), the most frequent valve disease, is associated with both. Severe MR complicated by AF is associated with excess mortality (6), but recent US guidelines appear to de-emphasize its role in surgical indications. Sudden cardiac death (SCD) due to VAs in patients with severe degenerative MR (1.8% per year) in excess to that in the general population has been a rational for early surgery, but recently malignant mitral valve prolapse (MVP) even without MR has been a focus of attention (7–10). Hence, the association of arrhythmias to all types of VHD has grown more complex to understand and manage.

Current guidelines reflect the underestimated relation between arrhythmias and VHD. Both atrial and VAs are poorly represented in AHA/ACC and ESC/EACTS guidelines on the managements of VHD (11, 12). These guidelines do not recommend Holter monitoring for arrhythmia detection. On the other hand, limited space is given to arrhythmias complicating VHD among guidelines for the management of AF and VAs (13). Moreover ACC/AHA and ESC/EACTS guidelines are divergent. AHA/ACC guidelines have recently eliminated AF as a trigger for surgery in patients with degenerative/primary severe MR whereas ESC/EACTS guidelines still consider it a class II and, for some authors, a class I recommendation for surgery (11).

Not only is management uncertain but mechanistic data on arrhythmia genesis in VHD remain rare. VA conceptualized as resulting from myocardial hypertrophy/fibrosis, has recently been attributed to the valve disease itself in the “arrhythmic MVP” (14, 15). Enlargement and fibrosis of the left atrium (LA) resulting from chronically increased filling pressures is considered a substrate for AF but the wide variation of LA alterations associated with AF leaves notable uncertainties (16). Furthermore, appropriate research on the role of recent diagnostic tools such as loop recorders, advanced electrophysiology and cardiac MRI warrants careful planning. Finally, although evidence of targeted therapies for VHD-related arrhythmia is accumulating, more research focused on indications and optimal timing of catheter ablation (CA) for atrial and VAs is required.

Therefore, it is crucial to review the available evidence to clarify management and plan appropriate trials for each type of VHD by identifying gaps in knowledge about prevalence, prognostic considerations and therapeutic options for atrial and VAs in VHD. We organized this review into chapters corresponding to the most common of VHD. Each chapter is further divided into sections addressing atrial and VAs. Separate sections successively describe the prognostic impact and current medical and interventional therapies of VHD-associated arrhythmias and highlight relevant questions for daily clinical practice. Conduction disturbances are beyond the scope of this review.

## Mitral Regurgitation

### Atrial Arrhythmias

The prevalence of AF in MR varies in the literature from 16 to 50%, depending on the method of detection, the definition of arrhythmia, and the populations studied (Table 1) (17, 18). The prevalence of AF in MR is higher in older patients; however, because of AF rarity in the younger general population, the excess AF risk is higher in younger (<65 yo) than in older patients (6, 43). The incidence of AF at 10 years is estimated to be 48% in patients with degenerative MR initially in sinus rhythm (SR) and managed conservatively (18). Whatever the mechanism of MR (flail leaflet, MVP), the incidence rate of AF is about 5% per year (18). Common atrial flutter is less frequent (~10% of the prevalence of AF), but both arrhythmias often coexist (44). MVP accounts for 1% of cardiac abnormalities diagnosed in young patients with common right-atrial flutter (45). Peri-mitral flutter is often associated with LA dilation or MR, but mostly occurs after catheter ablation (CA) of AF or MV surgery (46, 47).

**Table 1:** Atrial fibrillation in common sub-types of valvular heart disease: prevalence, impact on outcomes, and considerations related to the association of atrial fibrillation with valvular heart disease in current guidelines.

	**Mitral regurgitation**	**Mitral stenosis**	**Aortic stenosis**	**Aortic regurgitation**	**Tricuspid regurgitation**
**Prevalence**	16–50% (6, 17, 18)	40% (19)	Mild-to moderate AS: 2-17% (1, 20) Severe AS: 16–51% (21–23)	8–19% (24, 25)	Mild TR: 17–40%, Severe TR: 39–93% (26–34)
**Incidence**	5% per year (18)		1.2% per year (20, 35)	0.4% per year (36)	
**Cardio-vascular adverse events**	Significant impact on 5-year hospitalizations for congestive HF More bleeding and strokes after MR repair than for patient in SR (18)	Increased risk of systemic embolism (37)	In mild-to-moderate AS: increased risk of stroke and heart failure (38) New-onset AF after TAVI: associated with increased risk of bleeding, stroke, hospitalization for heart failure, and death (39)	Lower risk of stroke and systemic embolism than for MS or AS (4)	In patients undergoing isolated TV surgery, AF was identified as a determinant of major in-hospital complications (26)
**Prognosis**	Independent predictor (both paroxysmal and persistent AF) of increased all-cause mortality in conservatively and surgically managed degenerative MR (6, 18, 40)	Independent predictor of long-term adverse outcome after PMC (37, 41)	In mild-to-severe AS: increased all-cause mortality, regardless of symptoms and initial conservative or surgical management (20) In severe aortic stenosis: increased all-cause mortality (42) In severe AS undergoing TAVI: AF-associated increased mortality at 1 year (21, 39)	Increased risk of mortality in both conservatively and surgically managed patients (24, 25)	Increased risk of mortality (34)
**Current guidelines**	2021 ESC/EACTS: Surgery should be considered in asymptomatic patients with preserved LV function and AF as a class IIa indication (11) 2020 ACC/AHA (12): not cited	2021 ESC/EACTS: PMC may be considered (class IIa) for asymptomatic patients with new onset AF (11) 2020 ACC/AHA: PMC may be considered (class IIb) in asymptomatic patients with new onset AF (12)	Not cited	Not cited	Not cited

*ACC, American College of Cardiology; AF, atrial fibrillation; AHA, American Heart Association; AS, aortic stenosis; AR, aortic regurgitation; ESC, European Society of Cardiology; EACTS, European Association of Cardio-Thoracic Surgery; HF, heart failure; LAV: left atrial volume; MR, mitral regurgitation; MS, mitral stenosis; PMC, percutaneous mitral commissurotomy; SR, sinus rhythm; TAVI, transcatheter aortic-valve implantation; TR, tricuspid regurgitation; TV, tricuspid valve; VHD, valvular heart disease*.

#### Prognostic Impact of Atrial Arrhythmia

It is generally accepted that patients with long-standing degenerative MR develop AF *via* LA volume and pressure overload, progressive atrial fibrosis, and dilation, resulting in electroanatomical remodeling (48). Abnormal wall stress from the prolapsing leaflets and expanding mitral annulus are considered to be factors that promote atrial interstitial fibrosis, which is a recognized precursor of AF (16). Once AF has become established, it induces a vicious circle of self-perpetuation through LA enlargement (49). Persistent and permanent AF are predominant patterns observed in degenerative MR (50), with considerable impact on mortality, but paroxysmal AF diagnosed by ECG is already associated with impaired outcome vs. SR (6), raising the issue of its detection by more sensitive methods. Fibrosis increases LA stiffness, affecting cardiac filling and output with heightened risk of fibrotic atrial cardiomyopathy (48). Preoperative AF, even after surgical MR repair is associated with 50% higher relative risk of hospitalization for heart-failure (40), and excess mortality (6, 40, 51), emphasizing the importance of early detection of the LA precursors of arrhythmia, of paroxysmal arrhythmia and of the consideration of early surgery.

#### Management of MR With Atrial Arrhythmia

It is now accepted that AF detected during the clinical course of severe degenerative MR, even if paroxysmal, should trigger rapid consideration of surgery (6, 18, 52–54). Consequently, according to the 2021 ESC/EACTS Guidelines for the management of VHD, presence of AF (persistent or paroxysmal) should lead to the consideration of surgery for asymptomatic patients with severe MR and preserved LV function as a class IIa indication (11). Left atrial enlargement and advancing age are well-recognized precursors of AF in severe MR (18). Recent studies demonstrated that left atrial enlargement is strongly associated with excess mortality in patients still in SR with severe primary MR (51, 55, 56). Consequently, according to the 2021 ESC/EACTS guidelines, surgical mitral valve repair should be considered in low-risk patients with significant LA dilatation (volume index ≥ 60 ml/m^2^ and/or diameter ≥ 55 mm) regardless of symptomatic and rhythmic status when performed in a Heart Valve Center and a durable repair is likely (11).

Rhythm control is of recognized importance in the management of AF patients (13). However, data from the ORBIT-AF (Outcomes Registry for Better Informed Treatment for AF) showed that only 25% of patients with VHD and AF benefited from a rhythm control strategy and 10% underwent CA (57). Catheter ablation of AF patients with severe MR, if planned, should not interfere with the decision and timing of surgical repair. Although recent data suggested that successful CA of AF in patients with moderate MR promotes reverse remodeling on mitral valve apparatus and improves MR, it is not yet demonstrated whether a rhythm-control strategy is superior to a rate-control strategy on clinical outcome in non-severe MR (Table 2 and Supplementary Table 1) (58, 59). Therefore, AF management in patients with non-severe primary MR currently follows the general principles for patients with non-valvular AF because no data are available on the benefit of CA in the specific setting of non-severe MR (13, 60). In patients with AF and severe primary MR, concomitant surgical ablation of AF during mitral surgery should be considered (Class IIa 2021 ESC/EACTS Guidelines, class I by 2020 ACC/AHA and 2017 Society of Thoracic Surgeons Guidelines) (11–13, 61) but is underutilized and performed mainly by high volume mitral surgeons (62). Indeed, a beneficial effect of maze procedures associated with MV repair or replacement for degenerative MR, with persistent or permanent AF, has been demonstrated in randomized trials showing considerably higher maintenance of SR (70 vs. 37% at 1 year) than for patients treated with MV surgery alone (63–65). Pulmonary vein isolation for paroxysmal AF or surgical biatrial maze procedure should be considered in persistent AF (11, 12). Extensive biatrial lesions using bipolar radiofrequency or cryo-energy procedures performed during valvular surgery are associated with higher risk of pacemaker implantation, ~20% (63, 66).

**Table 2:** Gaps in evidence in the management of atrial fibrillation and ventricular arrhythmias coexisting with particular subtypes of valvular heart disease.

	**Mital regurgitation**	**Mitral stenosis**	**Aortic stenosis**	**Aortic regurgitation**	**Tricuspid regurgitation**
Atrial fibrillation	- Clarify the interest of early detection and therapy of silent AF in primary MR - Define the place and optimal timing of CA in the clinical course of MR - Determine the indications and results of hybrid ablation of AF in patients with severe degenerative MR undergoing surgery - Analyze the impact of AF on the results of new percutaneous valve repair and valve implantation techniques in patients with primary MR - Determine the impact of mitral valve interventions (surgery and catheter interventions) on AF/atrial tachycardias	- Determine the impact of PMC on AF recurrence - Define the place and optimal timing of CA of AF in significant MS - Define the impact of preoperative AF burden and LA volume on the results of adjunctive surgical ablation of AF	- Define whether AF should be considered in decision-making process in asymptomatic patients with severe AS - Determine the impact of AVR on outcomes for patients with AS in AF - Define the place and timing of CA for AF for patients with AS	- Determine the optimal timing and methods of AF ablation for patients with AR considered for surgery - Define the place of AF within the decision-making process - Evaluate the results of AVR for patients with severe AR in AF	- Determine the independent - Prognostic impact of AF - Determine whether AF should be considered in decision making process in severe TR - Determine the impact of TV interventions on outcomes in patients with AF - Determine the impact of the rhythm control interventions on the progression of TR
Ventricular Arrhythmia	- Define the optimal approach for risk stratification for patients with MVP, including the prognostic role of cardiac MRI and electrophysiological studies - Define the place of CA and cardiac surgery in arrhythmic MVP - Define the role of ICD for the primary prevention of SCD in arrhythmic MVP	- Define the prevalence, characteristics, and prognostic impact of VA - Define the place of ablative therapies	- Elucidate the physiopathology of VA in AS - Determine the sites of origin of VA in severe AS and the reversibility of multifocal PVC after AVR - Define the role and timing of CA for VA in severe AS - Determine the impact of early VTs following AVR on the risk of further recurrences and mortality	- Define the prognostic impact of VA in AR - Determine the sites of origin of VA in AR and its reversibility after AVR - Define the role and timing of CA for VA in severe AR	- Define the prevalence, characteristics, and prognostic impact of VA

*AF, atrial fibrillation; AS, aortic stenosis; AR, aortic regurgitation; AVR, aortic-valve replacement; CA, catheter ablation; ICD, implantable cardioverter defibrillator; MR, mitral regurgitation; MS, mitral stenosis; MVP, mitral-valve prolapse; PMC, percutaneous mitral commissurotomy; TR, tricuspid regurgitation; TV, tricuspid valve; VA, ventricular arrhythmia*.

Current knowledge on adjunctive LA appendage closure (LAAC) at the time of VHD surgery is growing but limited (67, 68). Results are promising in terms of reduction of embolic events in patients with a high burden of preoperative AF (69). One study focusing on the results of LAAC during mitral valve surgery demonstrated that LAAC was associated with fewer cerebrovascular events, but this benefit was seen only with concomitant surgical AF ablation (68). Another study comparing effects of the resection vs. preservation of the LAA during the maze procedure in conjunction with mitral surgery resulted in no significant differences in stroke-free survival and freedom from AF (70). Two recent metanalyses in populations of open cardiac surgery, not focused on mitral surgery, showed an association between LAAC and lower mortality (67, 69). Based on this data, current ACC/AHA guidelines consider this technique during valve surgery for patients with AF or atrial flutter (class IIa recommendation). This procedure is also recommended in the ESC EACTS guidelines for patients with AF and a CHA2DS2VASc score ≥ 2 undergoing valve surgery (class IIa recommendation) (11, 12).

There is an increasing emphasis on CA to prevent AF occurrence after mitral surgery. New-onset AF identified in 19-23% of patients 10 years after surgical correction of MR independently predicts subsequent stroke, heart failure and morbidity (6, 71). CA is estimated to be a good option (61% of AF-free at 1 year) for patients with mechanical mitral prostheses (72). Although AF is the predominant arrhythmia after valvular surgery, macro-reentrant atrial tachycardia may result from surgical incisions (73). Right atrial macro-reentrant circuits, cavo-tricuspid or incisional, are frequent after MV surgery (46, 74). Conversely, mitral annular flutter predominates after maze procedure (46, 75). Mapping and effective ablation of such tachycardias is considered to be feasible and safe, regardless of the presence of a prosthetic valve (75).

#### Considerations for Anticoagulation Therapy

Valvular AF is intended to imply rheumatic VHD with mitral stenosis (MS) or mechanical heart valves, and is considered contraindicating non-vitamin K oral anticoagulants (NOACs) (Table 3) (13). In native primary MR, NOACs are recommended in preference to vitamin-K antagonists (VKAs) for patients with AF, as class Ia (2020 ACC/AHA and 2021 ESC/EACTS) recommendation (11, 12).

**Table 3:** Considerations for anticoagulation therapy in patients with VHD and AF according to the ESC/EACTS and AHA/ACC guidelines.

	**ESC/EACTS guidelines**	**AHA/ACC guidelines**
MR, AS, AR	In native primary MR, AS and AR NOACs are recommended in preference to VKAs for patients with AF, as class Ia recommendation (11)	In native valve heart disease a NOAC is an effective alternative to VKA anticoagulation and should be administered on the basis of the patient's CHA2DS2-VASc score (class Ia recommendation) (12)
MS	Patients with moderate to severe MS and AF should be kept on VKA treatment, and NOACs are not recommended in this setting though recent data highlighted their potential efficacy (11)	In patients with rheumatic MS and AF anticoagulation with a VKA is indicated (12)
General considerations Flutter After bioprosthetic valve replacement After mechanical valve replacement Bridging	Valvular AF implies rheumatic VHD with MS or mechanical heart valves, and is considered contraindicating NOACS (11, 13) Anticoagulant therapy for atrial flutter is recommended based on the same risk score (CHADS2-VASC) as that used for AF (13) VKA therapy with target INR 2.5 is recommended for the first 3 months regardless of the heart rhythm status, but after that period NOACs appear equivalent (2020 ACC/AHA) or should be considered over VKA (2021 ESC/EACTS) to prevent embolisms due to AF (11) Target INR should be based upon prosthesis thrombogenicity and patient-related risk factors and AF is considered as one of patient-related risk factor requiring higher target INR for mechanical protheses (11) Bridging of OAC when interruption is needed in patients with valve replacement or repair is recommended for AF with a CHA2DS2-VASc score ≥ 3 for women or 2 for men, and in AF patients with significant MS (11)	Valvular AF implies rheumatic VHD with MS or mechanical heart valves, and is considered contraindicating NOACS (12) Anticoagulant therapy for atrial flutter is recommended based on the same risk score (CHADS2-VASC) as that used for AF (12) VKA therapy with target INR 2.5 is recommended for the first 3 months regardless of the heart rhythm status, but after that period NOACs appear equivalent. VKAs in patients with new-onset AF ≤ 3 months after bioprosthetic AVR (class IIa) and NOACs, administered on the basis of the patient's CHA2DS2-VASc score, in patients with AF > 3 months after a bioprosthetic valve (12) Anticoagulation with a VKA is recommended. For patients with an additional risk factor for thromboembolism including AF previous thromboembolism, LV dysfunction, hypercoagulable state or older-generation prosthesis anticoagulation with a VKA is indicated to achieve an INR of 3.0 (12) For patients with bioprosthetic heart valves or annuloplasty rings who are receiving anticoagulation for AF, it is reasonable to consider the need for bridging anticoagulant therapy around the time of invasive procedures on the basis of the CHA2DS2-VASc score weighed against the risk of bleeding (12)

*ACC, American College of Cardiology; AF, atrial fibrillation; AHA, American Heart Association; AS, aortic stenosis; AR, aortic regurgitation; ESC, European Society of Cardiology; EACTS, European Association of Cardio-Thoracic Surgery; INR, international normalized ratio; MR, mitral regurgitation; MS, mitral stenosis; NOAC, non-vitamin K oral anticoagulants; OAC, oral anticoagulation therapy, VHD, valvular heart disease; VKA, vitamin-K antagonists*.

Bridging of oral anticoagulation therapy (OAC), when interruption is needed, is recommended in patients with mechanical prosthetic heart valve, AF with a CHA2DS2-VASc score ≥ 3 for women or 2 for men, acute thrombotic event within the previous 4 weeks and high acute thromboembolic risk (11).

VKA therapy with target international normalized ratio (INR) 2.5 is recommended for the first 3 months after bioprosthetic mitral-valve replacement (MVR), regardless of the heart rhythm status, but after that period NOACs appear equivalent (2020 ACC/AHA) or should be considered over VKA (2021 ESC/EACTS) to prevent embolisms due to secondary AF (11, 12, 76). Anticoagulant therapy for atrial flutter is recommended based on the same risk score (CHADS2-VASC) as that used for AF (13, 60).

### Ventricular Arrhythmia

MVP prevalence in the general population is estimated between 1.7 and 2.4% (1, 2), presenting often with PVCs (77). The yearly incidence of SCD in patients with MVP has been estimated to be 0.2-0.4% and up to 1.8% per year in severe MR due to leaflet flail (8, 9, 76). “Arrhythmic MVP” without severe MR has been suggested as an underestimated cause of SCD in young adults (78, 79), although patients with MVP and proven VAs are often in their 60s (80).

#### Prognostic Impact of Ventricular Arrhythmia

**Figure 1:**
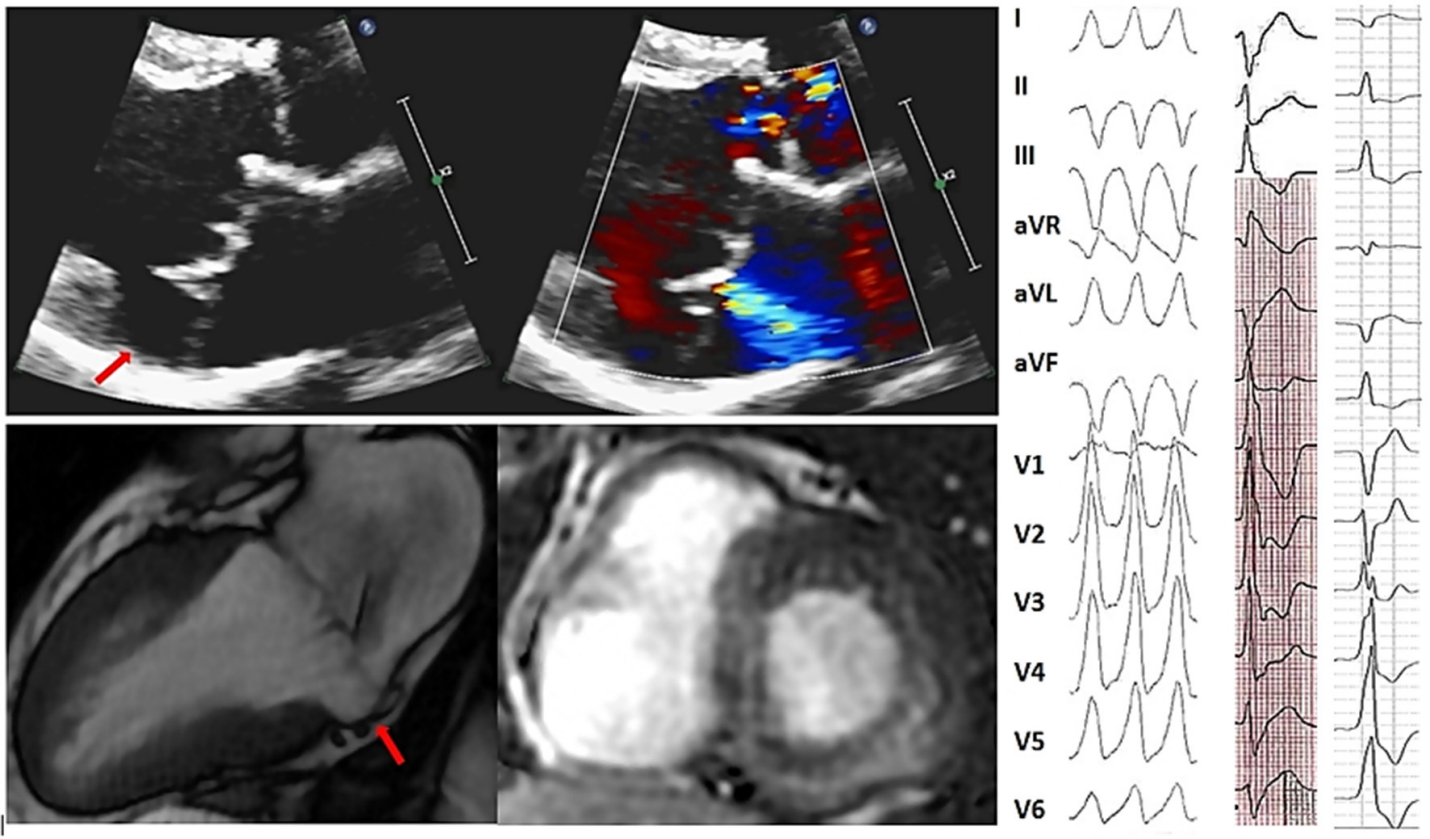
Representative of bileaflet mitral valve thickening and prolapse coexisting with mitral annular disjunction and myocardial arrhythmogenic substrate and 12-lead surface ECG illustrating multifocal ventricular arrhythmias in the same patient. Sixty-nine year old man without history of coronary artery disease referred for work-up of episodes of syncope. Bidimensional echocardiography, parasternal long-axis view at end-systole (upper panels) showed a bileaflet mitral valve thickening and annular disjunction (red arrow). Doppler evaluation showed a mild mitral regurgitation due to a bileaflet myxomatous mitral valve prolapse. Cardiac MRI cine imaging in two-chamber view (right bottom) identified a 11 mm large mitral annular disjunction localized to the posterior left ventricular wall and basal short-axis late gadolinium—enhanced (LGE) cardiac MRI (middle bottom) demonstrated midwall LGE most prominent at basal inferior and inferoseptal wall consistent with intramural myocardial fibrosis. Sustained monomophic ventricular tachycardia (on the right) with a right bundle branch block and superior axis coresponding to the posterobasal left ventricular wall origin was induced at the time of electrophysiology study with programmed electrical stimulation. Frequent multifocal premature ventricular complexes of two different morphologies were also recorded: the first one manifesting right bundle branch block configuration, V5 transition and D2/D3 negative/positive discordance pointing toward the anterolateral papillary muscle and the second one with left bundle branch block morphology, V3 transition and inferior axis consistent with an outflow tract origin.

There is currently a renewed interest in the study of this association of MVP with life-threatening VAs and this has led to the development of new concepts. Clinical presentation of “arrhythmic MVP” is often for palpitations and rarely syncope (79), but in patients who developed cardiac arrest, syncope was a frequent preceding symptom (81). While the initial focus was on bileaflet MVP as the main culprit (78), “arrhythmic MVP” is more specifically characterized with severe myxomatous disease, marked redundancy and mitral annular disjunction on the morphological side, independent of the degree of MR in the functional side, and with frequent inverted T-waves in the inferior leads and frequent PVCs by ECG interpretation (80, 81). Typically, patients with bileaflet MVP display more non-sustained VTs and a higher PVC burden on Holter monitoring, than those with normal MV, with a changing ECG morphology suggestive of the origin from the outflow tract, papillary muscle, or fascicular sites (78). Fibrosis localized to the posteromedial papillary muscle and infero-basal LV wall, identified using contrast-enhanced cardiac magnetic resonance (MRI) and in pathological studies has been highlighted as a potential myocardial source of VA that complicates MVP (79). However, severe VAs were rarely documented before SCD (9), possibly due to the scarcity of Holter-monitoring performed but patients who present with VA on monitoring incur a progressive but significant excess mortality (80). Severe VAs defined as non-sustained VT ≥ 180 beats/min or a proven history of sustained VT/VF are infrequent (9%) but associated with excess mortality subsequent to their diagnosis (80). Mitral annular disjunction (MAD) in the context of MVP can be diagnosed by cardiac MRI or echocardiography after cardiac arrest (Figure 1) (79, 81), and has been suggested a harbinger of such events but recent outcome data demonstrate that MAD is indeed associated with secondary development of arrhythmias but during the first 10 years is not associated with excess mortality and should not in and by itself lead to risky electrophysiological interventions (82). The link MAD-VA is hypothesized to be secondary to local fibrosis induced by the tension generated by such ample prolapse (15). More research is needed in order to establish the SCD-risk stratification algorithm in arrhythmic MVP (Table 2 and Supplementary Table 1).

#### Management of MR With Ventricular Arrhythmia

Most cases of severe organic MR are treated by surgery. Correction of the flail leaflet MR has been suggested to be associated with a lower risk of SCD (9, 80), but non-sustained VT on Holter-monitoring following MR repair may be a long-term predictor of SCD (83). After surgical MR repair or replacement, ICD implantation is indicated as class I recommendation for patients who satisfy implantation criteria according to current ESC Guidelines (84).

If mild to moderate MR is associated with PVCs or non-sustained VT, therapies that prevent SCD and provide symptomatic benefits should be considered. A history of palpitations or syncope or the detection of the phenotype associated with “arrhythmic MVP” (MAD, leaflet redundancy, and T-wave inversion) (80) should prompt Holter monitoring to identify VAs. Cardiac MRI should be discussed for risk stratification (79, 80). A predictive role of programmed ventricular stimulation to guide therapy for patients with VHD referred for syncope or VT has been suggested, but many uncertainties remain (85). An electrophysiological study is reasonable for patients with syncope if sustained-VT is suspected, based on symptoms or non-invasive assessment (84). Of note, frequent PVC can also result in LV remodeling in patients with less than moderate degenerative MR (86).

There are no specific data on beta-blockers efficacy for prevention of SCD in the setting of organic MR or MVP, but they are widely used as a first-line medical therapy for suppressing frequent symptomatic or complex PVCs and VTs. Other anti-arrhythmic agents may also be effective in the management of VHD-associated VA but must be used cautiously, given their potential to cause adverse events. Class I C sodium channel blockers should be avoided in cases of VHD with prior myocardial infarction (MI) or hemodynamically significant VHD (84).

In cases of non-responsiveness or a contraindication to antiarrhythmic agents, CA of symptomatic VAs originating from the papillary muscle in the presence of mild-to-moderate MVP has been reported to be effective (87–91). Catheter stability during the mapping and ablation of MVP-associated VA from the papillary muscle may be challenging. Significant progress has been recently made in CA techniques, such as the development of catheters with contact force sensors that improve safety and allow the creation of larger and deeper lesions. The use of new tools, such as intracardiac echocardiography that improve catheter positioning on the papillary muscles or cryoablation catheters that ensure better catheter stability during freezing, leads to higher acute and long-term success rates (90). An exhaustive list of studies reporting results of CA of VA in patients with VHD is presented in Supplementary Table 2. Current ESC and AHA/ACC/HRS guidelines for the management of patients with VAs and the prevention of SCD indicate CA of PVCs that trigger recurrent VF as a class I indication (84, 92). CA should be considered after failure or patient's preference of one or more antiarrhythmic agents as a class I (AHA/ACC/HRS) or IIa (ESC) recommendation for symptomatic patients with papillary muscle tachycardia. Regardless of MR severity, frequent PVC and non-sustained VT in the presence of predictors of mortality, such as MAD, leaflet redundancy, and T-wave inversion, should lead cardiologists to intensify beta-blocker therapy and discuss CA (84). Given the absence of specific guidelines for ICD implantation for the primary and secondary prevention of SCD in patients with primary MR, current recommendations for non-ischemic cardiomyopathy should be followed (84, 92).

## Mitral Stenosis

Mitral stenosis is characterized by obstruction of left ventricular inflow, with a chronic increase in LA pressure, resulting in progressive LA enlargement (93). The prevalence of AF in patients with MS is estimated to be 40% (19). MS-associated AF is associated with excess mortality and is a recognized predictor of systemic embolism (19, 37, 41). In contrast to other subtypes of VHD, the prevalence of MS in the western population of patients with AF decreased from 2-1.4% between 1998 and 1999 and 2009 and 2010 (1, 4). The hemodynamic consequences of MS reflect valve obstruction of the LA and pulmonary circulation but do not lead to LV dysfunction (93). Given the lack of data on VA in MS, we focus on MS-associated atrial arrhythmia.

### Prognostic Impact of Atrial Arrhythmia

The risk of the combined endpoint of stroke, systemic embolism, and all-cause mortality, is 4.2-fold higher in AF patients with MS as compared to patients with AF but without VHD (4). Percutaneous mitral commissurotomy (PMC) is recommended as the first-line treatment in severe symptomatic MS, regardless of the heart rhythm status (11, 12). In patients undergoing PMC, documented AF is a strong predictor of death, need for mitral surgery, and a redo PMC (41). In addition, in case of AF, a NYHA III-IV functional class is predictive of poorer late results after PMC (19). In asymptomatic patients, PMC should be considered for those with severe MS with a new onset of AF and favorable MV morphology (recommendation class IIa in ESC/EACTS guidelines or IIb in AHA/ACC guidelines) (11, 12). Indeed, new-onset AF is considered to be a turning point in the natural history MS triggering discussion of PMC (94).

### Management of MS With Atrial Arrhythmia

In patients with severe MS and recent onset AF, the 2021 ESC/EACTS guidelines recommend cardioversion soon after PMC (11). The use of antiarrhythmic agents to maintain SR is quite common around the world but with often disappointing results due to modest long-term efficacy or side effects (as described earlier) which require drug treatment interruption. Reported results of CA (multiple procedures) of AF in patients with mild MS in 5-year follow-up show maintenance of SR in only 45% for paroxysmal and in 26% for non-paroxysmal AF (95). Concomitant ablation of AF in MS surgery is recommended however, little is known about the impact of LA volume and preoperative AF burden on outcomes of such procedures (11, 12).

### Considerations for Anticoagulation Therapy

MS-associated AF is considered “valvular AF.” Accordingly, patients with moderate to severe MS and AF should be kept on VKA treatment, and NOACs are not recommended in this setting though recent data highlighted their potential efficacy (11, 12). VKA therapy to achieve should be prescribed after mechanical MVR (11, 12). Target INR should be based upon prosthesis thrombogenicity and patient-related risk factors and AF is considered as one of patient-related risk factor requiring higher target INR for mechanical protheses (11). VKA therapy should also be recommended for patients with AF for the first 3 months after bioprosthetic MVR, whereas NOACS should be considered over VKA for patients with AF after remote bioprosthetic MVR (11, 12, 96).

## Aortic Stenosis

### Atrial Arrhythmia

AS induces hemodynamic LV afterload increase, resulting in LV hypertrophy, chronically increased LV filling pressure, and LA enlargement, leading to increased AF risk (21). AF is, therefore, frequent in mild-to-moderate AS, with estimated prevalence of 17% and incidence of new-onset AF of 1.2% per year (20, 35). Although new-onset AF is often symptomatic, it has been identified in 20% of asymptomatic patients with severe AS (22). Conversely, AS is identified in 2-5% of patients with AF (4). The frequency of pre-operative AF in patients with AS referred for AVR is estimated to be between 16 and 35% (22, 38). The reported prevalence of pre-existing AF among TAVI studies ranges from 16% to as high as 51% (21, 23).

#### Prognostic Impact of Atrial Arrhythmia

In mild-to-moderate AS, AF is associated with increased risk of stroke and HF (35, 97). A pilot study that included patients with mild-to-moderate and severe AS suggested a significant impact of AF on mortality (20). In this study, patients in AF at AS diagnosis displayed higher mortality than those in SR (60 vs. 24%), irrespective of medical or surgical management. Although one study failed to demonstrate a link between AF and outcome in severe AS (22), a recent analysis of a large real-life cohort of patients with severe AS and preserved ejection fraction shows that associated AF is independently predictive of mortality, regardless of the symptomatic status (42). This study also reports in these patients with AF and severe AS, that AVR is associated with better survival compared to conservative management. Moreover, combining LAVI (left atrial volume index) with the presence or absence of AF is useful for the risk stratification (98). Patients with AF and LAVI > 50 ml/m^2^ incur the highest risk of death while those in SR with LAV ≤ 50 ml/m^2^ have the lowest mortality rate. Interestingly, patients with AF and LAV ≤ 50 ml/m^2^ show the same prognosis as those in SR with LAVI > 50 ml/m^2^—thus forming an intermediate-risk group (98). These data suggest that, in patients with asymptomatic severe AS, referral for AVR should be ideally discussed before overt episodes of AF and when LAVI is still below 50 ml/m^2^. Several studies have demonstrated a negative impact of preexisting AF on prognosis in patients undergoing TAVI. In patients with known pre-operative AF, 1-year post-TAVI overall mortality is significantly higher (ranging from 18 to 36%), as compared to patients without documented AF (8-25%). Furthermore, preoperative AF has been identified as an independent predictor of perioperative and 5-year mortality in severe AS with low ejection fraction ( ≤ 35%) undergoing surgical AVR (38). However, more data on AF impact on outcomes of patients with severe AS are still needed to integrate heart rhythm into the clinical decision-making process.

Furthermore, recent data emphasize a prognostic impact of new-onset AF following AVR. In a recent study that included 72,660 patients who underwent TAVI, new-onset AF during the follow-up was associated with two-fold mortality risk than no-AF and with higher risk of mortality, bleeding, stroke and hospitalization for HF than pre-existing AF (39). Of note, the follow-up period in the above-cited TAVI studies were limited to 12 months (23, 39, 99).

#### Management of AS With Atrial Arrhythmia

In patients with severe AS, AF occurrence can trigger hemodynamic decompensation and lead to poor outcomes. Beta-blockers are used for AS as first-line rate-controlling agents for permanent AF (13, 100).

Little is known about CA of AF efficacy and timing in AS (Table 2 and Supplementary Table 1). In clinical practice, AVR is recommended for symptomatic severe AS, regardless of heart rhythm (11, 12). Surgical pulmonary vein isolation or maze procedure for paroxysmal or persistent AF during valvular surgery is considered a class IIa indication (11, 12). For symptomatic AF patients with mild-to-moderate AS, rhythm control with CA should be discussed.

#### Considerations for Anticoagulation Therapy

AS-associated AF is not considered as “valvular AF.” Accordingly, NOACs are considered to be a good alternative to VKAs in patients with AS and AF as a class Ia recommendation (2020 ACC/AHA) or a class IIa recommendation (2021 ESC/EACTS) (11, 12). In patients with AF and bioprosthetic AVR, ESC/EACTS recommendations place NOACs over VKAs after the initial period of 3 months following surgery (11). 2020 ACC/AHA guidelines recommend VKAs in patients with new-onset AF ≤ 3 months after bioprosthetic AVR (class IIa) and NOACs, administered on the basis of the patient's CHA2DS2-VASc score, in patients with AF > 3 months after a bioprosthetic valve (12).

### Ventricular Arrhythmia

Myocardial hypertrophy and fibrosis due to increased LV systolic afterload constitute a structural substrate for VAs (14). Frequent CAD coexistence in the elderly, often with ischemic scar predisposes patients with severe AS to VAs. Potential source of idiopathic VAs originating from aortic cusps attributed to muscular fibers extending from the outflow tract, is not established in AS (101, 102). In studies that analyzed VAs in symptomatic severe AS before TAVI using 24-h Holter monitoring, complex PVCs were present in 48% and non-sustained VT in 9-29% (103, 104). A significant decrease in higher grade VA (pairs from 17 to 5%, non-sustained VTs from 9 to 2%) at 12 months after TAVI was observed. Atrio-ventricular (AV) nodal or His-Purkinje conduction delay which is commonly seen after TAVI, can create the right milieu for development of bundle branch VT (105). In patients undergoing surgical AVR available data show the early incidence of sustained VTs to be low (≈1%) (106). Outflow tract non-sustained VT was reported in 5% of patients with prior surgical AVR at 10-year follow-up (107). Patients with prior AVR in the absence of known MI account for 4% of cases referred for CA of recurrent VT (106).

#### Prognostic Impact of Ventricular Arrhythmia

VAs in AS are clinically considered to be a marker of impaired ventricular function and a harbinger of syncope and SCD (108). In non-operated symptomatic patients with severe AS, SCD is a frequent mode of death (14). SCD is much less frequent (~1% per year) in asymptomatic patients with severe AS (109). However, mechanisms of SCD remain undefined between VAs, AV block or acute HF. Calcifications infiltrating the junction His bundle-left bundle branch can predispose to high degree AV block or bundle-branch VT (110). Repair of congenital AS reduces the risk of SCD but the incidence of SCD still reaches 20% at thirty-year follow-up (111).

#### Management of AS With Ventricular Arrhythmia

Mild-to moderate AS patients manifesting palpitations or syncope evocative of arrythmia should undergo a diagnostic work-up that includes heart-rhythm monitoring and treadmill test (12, 92, 112). The presence of VAs is not considered by current guidelines to be an indication for AVR in severe AS (11, 12). In the presence of identified VAs originating from the LV, cardiac MRI can be used to diagnose fibrosis or ischemic scar. Programmed ventricular stimulation is useful for patients with previous MI or other scar-related conditions admitted for syncope that remains unexplained after non-invasive evaluation and when tachycardia is suspected (113).

Specific data on medical and ICD therapy of VA in patients with AS is lacking. Beta-blockers for primary prevention of SCD have not been specifically evaluated in AS (114), but can be used for suppressing symptomatic VAs. In contrast, sodium channel blockers antiarrhythmic agents are contraindicated with prior MI, often observed in AS (84). Amiodarone can be an effective adjunctive therapy for recurrent non-sustained VT but efficacy for preventing SCD is unproven. ICD implantation in patients with VHD (~7% of all secondary preventions) provides appropriate protection, with similar rate of ICD shocks and mortality vs. patients with CAD or dilated cardiomyopathy (115, 116). Associated cardiac resynchronization therapy may be considered (117). Accordingly, ICD therapy is recommended following general principles (84, 92).

**Figure 2:**
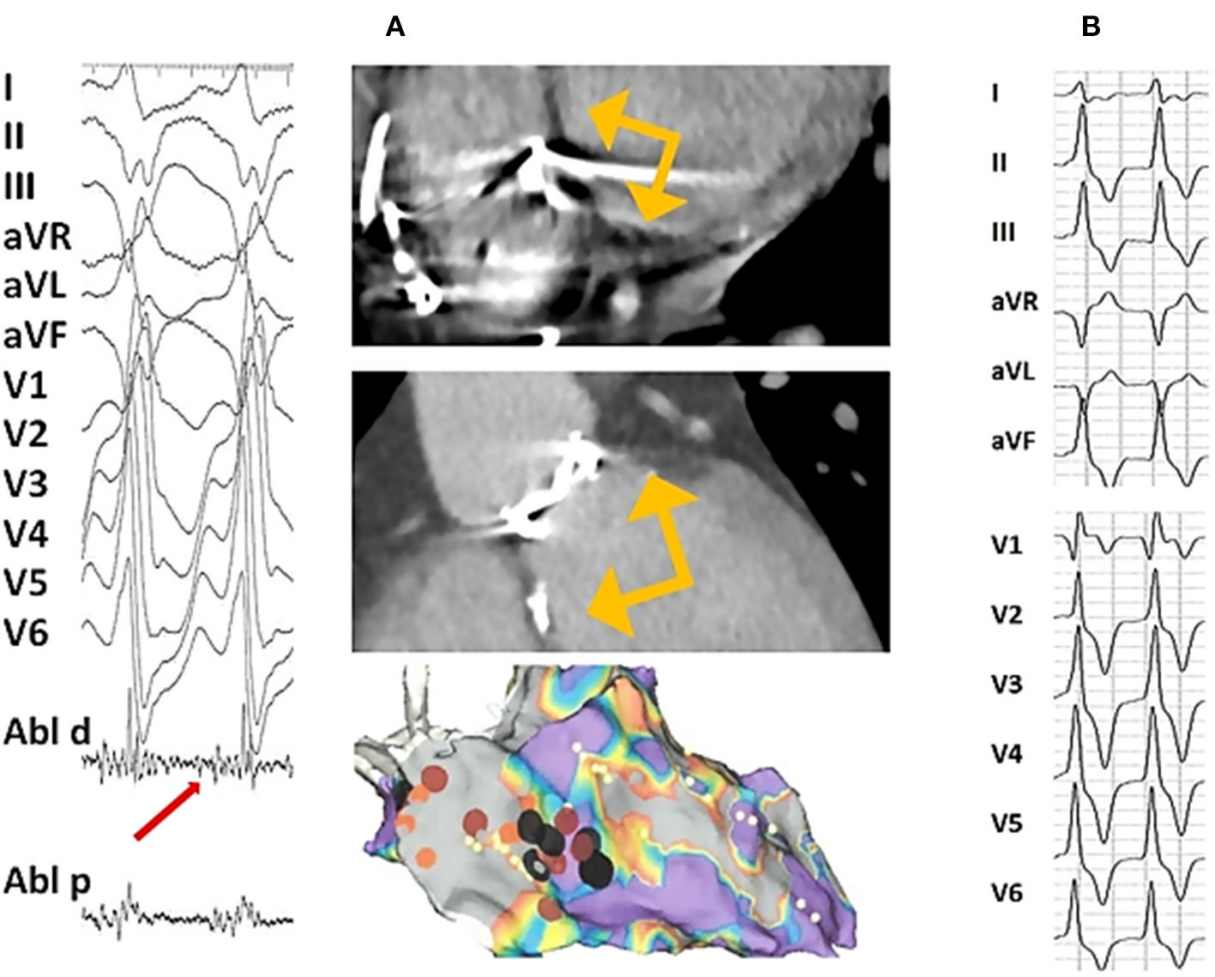
Twelve-lead surface ECG of ventricular tachycardia following aortic valve replacement and representative of CT scan and electroanatomic substrate: **(A)** In a 23 year-old man with congenital aortic stenosis treated with balloon aortic valvotomy followed by surgical aortic valve replacement with mechanical Carbomedics 21 prosthesis at the age of 7 years and implanted with a double chamber pacemaker for post-operative complete atrio-ventricular heart block. At the age of 23 years, he was admitted for a sustained symptomatic ventricular tachycardia leading to upgrading his pacemaker to implantable cardioverter defibrillator. Cardiac CT scan (on the top : coronal oblique left ventricular outflow tract and basal short-axis views) performed prior to electrophysiology study showed a periaortic left ventricular myocardial thinning localized to anteroseptal, inferoseptal and inferobasal LV segments (yellow arrows) consistent with a periaortic scar. Bipolar endocardial electroanatomic map (on the bottom, antero-posterior view) displayed periaortic inferoseptal low-voltage abnormal area (in gray). The clinical monomophic VT with a right bundle branch block and superior axis configuration was induced at the time of electrophysiology study with programmed electrical stimulation. The earliest recorded activation signal (50 ms to the onset of the surface QRS recorded on distal tip of the ablation catheter, red arrow) identified at the edge of the low voltage inferoseptal area was targeted with ablation (black dots) allowing for VT interruption. **(B)** A 18 year-old man with congenital severe bicuspid aortic valve stenosis leading to aortic valvuloplasty at the age of 5 and 16 years followed by surgical aortic valve replacement with mechanical 21 St Jude prosthesis at the age of 17 years. One year after aortic valve replacement an asymptomatic non-sustained monomorphic VT was recorded on a 24 h Holter and exercise treadmill test. 12-lead ECG displayed a Rs morphology in D1, qR pattern in V1 and a lack of precordial transition suggesting aortomitral continuity source.

After AVR, electrophysiology studies for VT demonstrated that most cases were consistent with macro-reentrant mechanism in relation to LV periaortic low-voltage area (see case illustration Figure 2) or to a bundle-branch reentrant VT (106, 118). Endocardial CA post-surgical AVR for VT is safe and effective but has not been tested post-transcatheter AVR (Supplementary Table 2) (106, 118). Whether periaortic scar preexists as a non-ischemic process or is consequent to surgery is unresolved. ESC Guidelines for management of patients with VA and prevention of SCD recommend electrophysiological study with standby CA in patients who develop VT following valvular surgery to identify and perform CA in case of bundle branch re-entry VT (class IIa recommendation) (84).

## Aortic Regurgitation

AF is observed in 8 to 19% of patients with severe AR (24, 25). AR prevalence among patients with AF increased from 1.2 to 2.3% between 1998 and 2010 (4). AF associated with AR is a strong independent predictor of mortality under both conservative (HR: 4.53) and surgical management (HR: 3.28) (24). There is no specific data about the prognostic impact of AVR on patients with AF and AR.

Preoperative Holter recordings showed frequent PVCs in 22% and non-sustained VT in 12% related to the severity of LV remodeling, but link to outcome is uncertain (119). Extreme ventricular dilatation in patients with severe AR may be linked to SCD but the very few cases reported leave considerable doubt (36). Poor LV function appears more specifically linked to SCD in severe AR (120). General principles of guidelines for the management of patients with VA and the prevention of SCD should be applied in the setting of patients with AR.

## Tricuspid Regurgitation

### Atrial Arrhythmia

The prevalence of AF varies in the literature from 17 to 40% for mild and from 39 to 93% for severe TR, depending on the populations studied (26–34). Tricuspid regurgitation induces chronic increase in right ventricular filling pressures, and ultimately right atrium (RA) dilation and fibrosis. Therefore, TR can lead classically to AF through unfavorable adaptation of the RA to the pressure and volume changes. However, AF itself, through its link to RA dilation resulting in tricuspid annular enlargement, can also lead to TR. This predominant mechanism of RA remodeling and marked annular dilation occurs mainly in elderly patients with high prevalence of AF defining a group of particular interest. Anterior-posterior diameter and annular area, are significantly larger in AF-related TR, whereas tenting volume and tethering angles are significantly higher in other causes of functional TR (34). The RV dilation in AF-related TR is secondary to TR. Then, both RA and RV enlargement can lead, in turn, to the worsening of TR, creating a kind of vicious circle. This particular form of TR has been recently a focus of attention and called by some authors “atrial” TR (121–123). Atrial TR is not rare, concerning from 6 to 9% of moderate to severe TR, and is characterized by the absence of structural left valve disease, left ventricular dysfunction, pulmonary hypertension, or overt cardiac cause. Nevertheless, both AF and TR can cause RA enlargement and it is often difficult for the clinician to determine whether AF is a cause or a result of TR. Moreover, AF can beget TR not only in isolated but in all types of TR (33).

#### Prognostic Impact of Atrial Arrhythmia

Atrial fibrillation is strongly associated with the severity of TR (27, 31). Moreover, AF may contribute to a rapid progression of TR severity (124). Indeed, patients with non-severe TR at baseline who experienced TR progression have larger left and right atrial volumes and a higher prevalence of AF (125).

Regardless of the heart rhythm status, at least moderate TR is independently associated with increased mortality in organic, functional or isolated TR and the excess mortality is higher in advanced stages of TR (28–30, 32, 34, 124). Although one study found an association of AF with worse outcome in isolated TR (33), the independent link between AF and excess mortality in severe TR has not been clearly demonstrated. Consequently, AF appears to be one of factors associated with advanced TR but not the major one responsible for increased mortality and AF is not cited in current guidelines on the management and decision making of TR (11, 12). In patients undergoing isolated tricuspid valve surgery, AF was identified as a determinant of major in-hospital complications (26). A recent study analyzing predictors of adverse outcomes after transcatheter tricuspid valve repair demonstrated that patients with TR-associated AF had better outcome than patients with pulmonary hypertension or patients on dialysis (126). Actually, we need more information on the prognostic impact of AF in severe TR according the etiology. Today we possess sufficient data showing the link of AF with the progression of TR. Closer follow-up in order to detect AF and rhythm control strategy to contain annular dilatation and delay advanced stages of TR should be proposed (12, 121). Despite their prognostic implications, both TR and AF still remain underdiagnosed and more effort and research is needed to optimize management of patients with TR and AF.

### Ventricular Arrhythmia

Data on TR-associated ventricular arrhythmia are very scarce, focused on Ebstein anomaly. This congenital heart disease can be associated with single or multiple right-sided accessory pathways or Mahaim pathway generating reentrant atrioventricular tachycardia and the risk of life-threatening VA depending of the electrophysiological properties of accessory pathway (127). Ablation of these arrhythmias can be performed safely in pediatric patients (127). Other types of congenital heart disease, with right heart volume overload such as atrial septal defect and repaired tetralogy of Fallot may also lead to VAs (128).

#### Difficulties of Assessment of the Functional Status of VHD Related to AF

**Figure 3:**
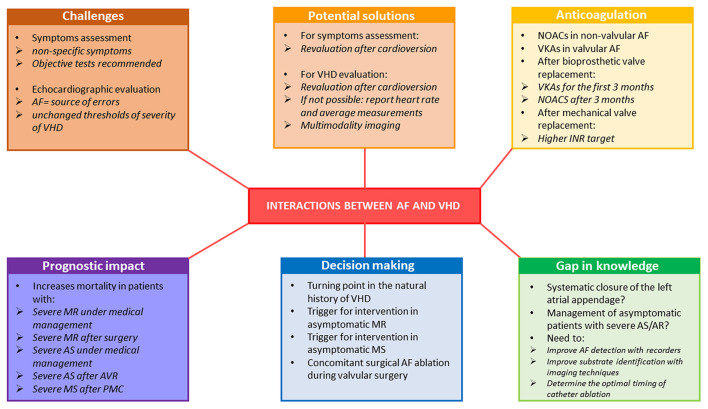
Representative of the interaction between AF and VHD in terms of diagnostic challenges and prognostic and therapeutic considerations. AF, atrial fibrillation; AR, aortic regurgitation; AS, aortic stenosis; INR, international normalized ratio; MR, mitral regurgitation; MS, mitral stenosis; NOAC, non-vitamin K oral anticoagulants; PMC, percutaneous mitral commissurotomy; VHD, valvular heart disease; VKA, vitamin-K antagonists.

Patients with VHD develop various symptoms such as exertional dyspnea, decreased exercise tolerance or HF, exertional angina, exertional syncope or presyncope and fatigue depending on the subtype of VHD (12). However, these symptoms are not specific for VHD (Figure 3). Exertional dyspnea may be attributable to hemodynamical consequences of valve disease but also to other cardiac conditions including arrhythmias which are notably prevalent in this population. Moreover, when AF is detected in the clinical course of VHD, it is not obvious whether this is a VHD-related heart rhythm disorder or a by-standing independent condition. Furthermore, all non-cardiac factors contributing to symptoms need also to be sorted out such as anemia or pulmonary disease.

The majority of patients with severe VHD are symptomatic when AF is present. Although the change in heart rhythm status does not always lead to symptoms, the loss of SR often reveals the progression of underlying VHD, may contribute to clinical worsening and be a turning point in the natural history of VHD. Given the subjective character and progressive development of symptoms, some patients may claim to be asymptomatic even in the presence of advanced VHD. In clinical practice, evaluation confirming the absence of symptoms such as exercise testing, when feasible, and serum B-type natriuretic peptide (BNP) measurement is recommended (11, 12). In apparently asymptomatic patients with severe AS, aortic valve replacement should be discussed when the exercise test is abnormal or the BNP level is >3 times normal regardless whether SR or AF is present (12). In the specific case of asymptomatic severe primary MR or MS, the presence of paroxysmal AF should lead to discuss surgery in MR and PMC in MS (11).

In symptomatic patients with severe VHD in AF, it is extremely difficult for the clinician to distinguish symptoms related to VHD from those related to AF. This difficult issue has not been studied in the literature and is not discussed in current guidelines (11, 12). In clinical practice, in patients with severe VHD associated with permanent AF, functional status is reevaluated after optimal rate control. However, the question of whether the patient's symptoms are mainly related to severe VHD or to AF is not taken into account in the decision making process in current guidelines and restauration of SR for reassessment of the severity of symptoms is not proposed (11, 12).

#### Difficulties of Assessment of Echocardiographic Severity of VHD Related to AF

**Figure 4:**
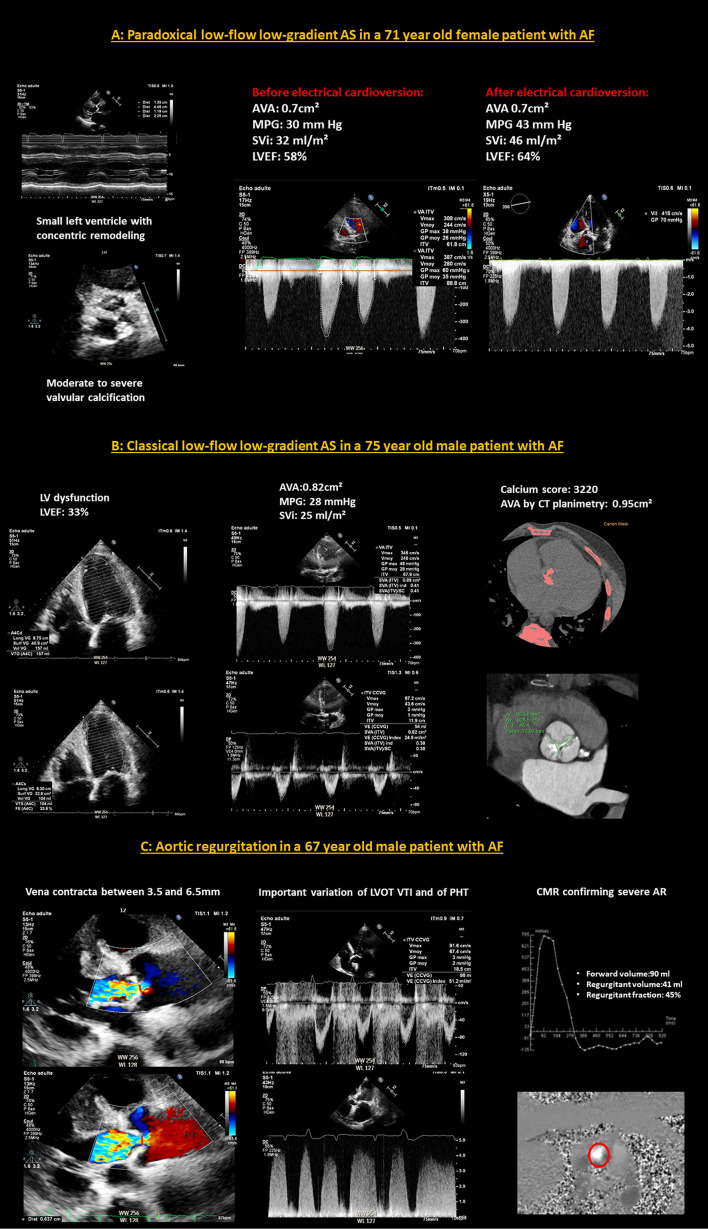
Examples of difficult cases of echocardiographic evaluation of aortic valve disease in patients with AF. **(A)** A case of paradoxical low-flow low-gradient AS in a 71-year-old female patient with AF. The left ventricle is small with concentric remodeling and preserved LVEF and the aortic valve presents moderate to severe calcifications. There is a clear variation of the aortic flow according to the cardiac cycles. The MPG is low, calculated at 30 mm Hg and the SVi is averaged at 32 ml/m^2^, in favor of a paradoxical low-flow, low-gradient AS. After electrical cardioversion, and the restoration of SR, the cycles no longer fluctuate, the SVi has normalized to 46 ml/m^2^ and the MPG is high at 43 mmHg. The AVA remained stable at around 0.7cm^2^ before and after cardioversion. **(B)** A case of classical low-flow low-gradient AS in a 75-year-old male patient with AF. There is a left ventricular dysfunction, with a LVEF estimated at 33%. The flows vary slightly according to the cardiac cycles. The MPG is low, averaged at 28 mmHg as well as the SVi which is estimated at 25 mml/m^2^. The cardiac CT-scan is in favor of a severe AS because the calcium score is very high at 3,220 and the aortic valve planimetry finds 0.95cm^2^. **(C)** A case of aortic regurgitation in a 67-year-old male patient with AF. It is difficult to distinguish between a moderate and a severe AR on echocardiography because of the marked variation of the parameters (vena contracta, LVOT VTI and PHT) between cardiac cycles. Evaluation by phase-contrast CMR, which is less disturbed by AF than echocardiography, leads to the conclusion of severe AR with a calculated regurgitation fraction of 45%. AF, atrial fibrillation; AR, aortic regurgitation; AS, aortic stenosis; AVA, aortic valve area; CMR, cardiac magnetic resonance; LVEF, left ventricular ejection fraction; LVOT VTI, left ventricular outflow tract velocity time integral; MPG, mean pressure gradient; PHT, pressure half time; SR, sinus rhythm; SVi, stroke volume index.

In clinical practice, AF complicates the evaluation of all VHD and makes their quantification more difficult because most of the criteria used routinely are impacted by the arrhythmia. However, despite the widespread association between AF and VHD, there is no specific data in the literature on this subject. Therefore, the severity thresholds of VHD are not different for patients evaluated in AF (129, 130). However, the echocardiographic quantification of VHD in AF requires special attention. European (129) and American Guidelines (130) do not specifically address this point and only mention the fact that measurements in patients in AF should be averaged over several cycles (5–10) and that some criteria should be interpreted with caution in patients in AF such as hepatic and pulmonary systolic vein flows that can be blunted by AF, resulting in a lack of specificity of these parameters in TR and MR assessment (129, 130). In clinical practice, the most crucial point is certainly that, for each parameter, whether it is a regurgitation or a stenosis, each measurement must be averaged over several cardiac cycles, with the least variation of R–R intervals and as close as possible to normal heart rate avoiding short diastoles. It is also important to report the heart rate at which gradients are measured for AS and MS evaluation and to be very careful in the interpretation of the continuity equation which can easily be mistaken (131). Thus, the quantitative evaluation of VHD is much more difficult in AF, which is a traditional limit of echocardiography and an important source of errors, which requires experience. At the slightest doubt, one should not hesitate to confirm the severity of VHD by other examinations such as a calcium score for AS, a cardiac MRI for regurgitation VHD or by cardiac catheterization in cases of persistent doubt (129–131). In particularly difficult cases, when there is doubt about the severity and a possible indication for intervention if the VHD is really severe, some clinicians propose to restore the SR, when possible, for example by electrical cardioversion, to give the opportunity to re-evaluate the echocardiography in SR and obtain a more reliable quantification. However, there is neither recommendation for this approach in the guidelines, nor data in the literature. Examples of difficult cases of echocardiographic evaluation of aortic valve disease in patients with AF are illustrated in Figures 4A–C.

#### Postoperative Atrial Fibrillation After Valvular Interventions

**Figure 5:**
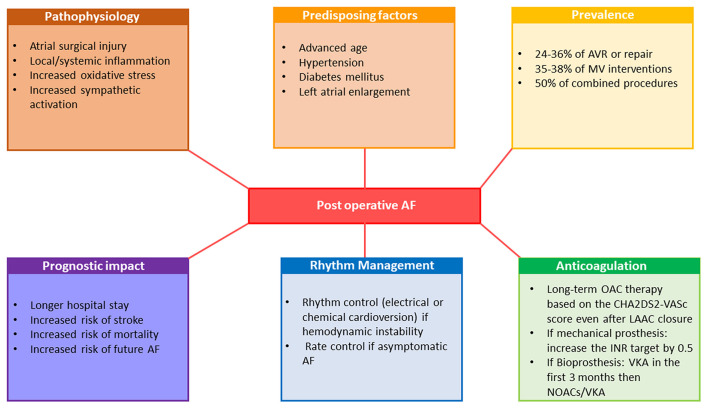
Pathophysiology, prognostic implications and management of postoperative atrial fibrillation. AF, atrial fibrillation; AVR, aortic valve replacement; INR, international normalized ratio; LAAC, left atrial appendage closure; MV, mitral valve; NOAC, non-vitamin K oral anticoagulants; OAC, oral anticoagulation therapy; VKA, vitamin-K antagonists.

AF is a common perioperative complication of cardiac valvular surgery (Figure 5). After exclusion of patients who have concomitant coronary revascularizations, postoperative (i.e., intrahospital AF) accounts for 24-36% of AVR or repair, 35-38% of MV interventions and for 50% of combined procedures (132).The risk of new-onset postoperative AF results from increased oxidative stress, local and systemic inflammation, atrial surgical injury and particularly from increased sympathetic activation and is higher in the presence of predisposing factors like advanced age, hypertension, diabetes mellitus and LA enlargement (133). Postoperative AF is associated with prolonged hospital stays, increased risk of stroke, and in-hospital and late mortality (134). Moreover, it has been demonstrated that in patients undergoing open heart surgery, postoperative AF carries an increased risk of future AF. Studies testing prophylactic use of corticosteroid agents, statins, colchicine, magnesium, betablockers, sotalol, amiodarone, n-3 polyunsaturated fatty acids, ascorbic acid and posterior pericardiectomy to prevent new-onset postoperative AF showed variable results (13, 135, 136). Of those, only betablockers and amiodarone were considered as having a beneficial effect and their perioperative use constitutes a class I recommendation in current ESC guidelines (13). Hemodynamic instability should lead physicians to consider perioperative AF as an indication of electrical cardioversion for restoration of SR while asymptomatic postoperative AF should initially be managed with rate control and anticoagulation (13).

Target INR for mechanical protheses in patients with AF are 0.5 higher than in patients in SR and varies between 3.0 and 4.0 depending on the type of protheses (11, 12). Given conflicting data about the safety and efficacy of NOACs in AF patients early after implantation of a bioprosthesis, the current guidelines still favor using VKA in the first 3 months after surgical or transcatheter bioprosthetic valve implantation (11, 12). However, NOACs should be considered over VKA after 3 months following surgical implantation of bioprothesis in patients in AF (class IIa recommendation) (11). For AF patients undergoing TAVI, OAC alone was non-inferior to OAC plus clopidogrel with respect to ischemic events (137). According the current guidelines, long-term anticoagulation therapy to prevent thrombo-embolic events should be considered as a class IIa recommendation in patients at risk for stroke with postoperative AF regardless of AF pattern and duration (13). Long-term anticoagulation therapy is also recommended in patients after AF surgery and LAAC, based on the patient's thrombo-embolic risk assessed with the CHA2DS2-VASc score, as a class I recommendation (13).

### The Way Forward—Action Plan

Seminal data suggest that arrhythmias are frequent in patients with VHD and may yield higher risk of cardiovascular complications and excess mortality. However, data are few and retrospective, implying that incidence, prevalence and outcome/management implications of clinical and subclinical arrhythmias remain undefined. Therefore, guidelines regarding detection and management of arrythmias in the context of VHD remain vague and limited. Hence, structured investigations are warranted to clarify pathophysiology, epidemiology, prognosis and treatment of VHD-associated arrhythmias.

Holter monitoring is not systematically recommended and diagnosis of arrythmias in VHD still remains at individual cardiologist discretion. Little is known about prevalence and patterns of arrhythmias, particularly transient or subclinical, in specific subtypes of VHD. Thus, yield and indications of cardiac monitoring, using 12-lead Holter monitoring and more recent diagnostic tools such as sensitive loop recorders, should be determined prospectively in various VHD. Establishing burden, type, sites of origin and severity of atrial and ventricular arrhythmias using these methods and exploiting large registries is a starting point for further appropriate prospective research that warrants careful planning.

Second, severity and prognostic implications of arrhythmias in patients with VHD will require large registries providing sufficient power to define clinical determinants and outcome implications accounting for well-defined covariates. The role of advanced electrophysiologic testing including programmed ventricular stimulation needs to be determined for complex clinical cases such as malignant MVP. Defining pathophysiology will require advanced imaging techniques, including cardiac MRI for the detection of ventricular fibrosis, hypertrophy and ischemic scars. Emphasis should be placed on quantifying the identified abnormalities such as fibrosis and to establish data-driven thresholds of severity for arrhythmogenic substrates. Similarly, identifying substrate for atrial arrhythmias will require imaging techniques aimed at overcoming uncertainties linked to wide variations of atrial alterations.

Pilot data suggest that targeted therapies for VHD-related arrhythmia may be effective, clinical trials will be required to define indications and optimal timing of electrophysiologic interventions in specific VHDs. While beneficial effect of surgical ablation of AF during mitral valve surgery has been demonstrated, more research is needed to define optimal lesion sets for improved outcome and to analyze comparative efficacy and timing of percutaneous AF ablation. Approaches to arrhythmias in each specific VHD type and stage (mild to severe), regarding timing and type of interventions will require well designed clinical trials.

While the risk of life-threatening VAs appears attenuated after valve surgery, the benefits and indications for ICD remains poorly defined. PVC ablation is more often performed recently, but it is unclear whether it is effective in improving quality of life or survival itself. Thresholds of PVC burden, type and severity for ablation therapy need to be defined as a preamble to clinical trials of this therapy.

Therefore, while data on arrhythmias in VHD may raise alarms, their paucity yields poorly understood clinical implications and underpin the present call for new research that may ultimately improve outcomes of the large segment of the population affected by VHD.

## Conclusions

Valvular heart disease is often associated with arrhythmia, which predisposes patients to a higher risk of cardiovascular complications and excess mortality. Both atrial and ventricular arrhythmias may contribute to clinical worsening and be a turning point in the natural history of VHD. Symptoms developed in patients with VHD are not specific and may be also attributable to other cardiac conditions including arrhythmias. Atrial fibrillation, the most common atrial arrhythmia, is an important source of errors in echocardiographic evaluation of VHD. Evidences on prognostic impact and treatment of VHD-associated arrhythmias based mostly on retrospective studies are growing but the guidelines regarding detection and management of arrythmias in the context of VHD remain limited. Postoperative AF complicating valvular interventions is frequent and many questions remain regarding its prevention and optimal management. Despite the known evidences, and undeniable recent progress in understanding of underlying mechanisms, the association of VHD with arrhythmias still remains underestimated and structured research on its pathophysiological genesis, diagnosis, prognostic impact and optimal management are warranted.

## Author Contributions

MK: conception and design of the research, analysis and interpretation of the data, and drafting of the manuscript. CC: critical revision of the manuscript for important intellectual content. YB: acquisition of data, analysis, and interpretation of the data. PL: critical revision of the manuscript. ME-S: critical revision of the manuscript for important intellectual content. CT: conception and design of the research, analysis and interpretation of the data, statistical analysis, critical revision of the manuscript for important intellectual content, and supervision. All authors contributed to the article and approved the submitted version.

## Conflict of Interest

The authors declare that the research was conducted in the absence of any commercial or financial relationships that could be construed as a potential conflict of interest.

## Publisher's Note

All claims expressed in this article are solely those of the authors and do not necessarily represent those of their affiliated organizations, or those of the publisher, the editors and the reviewers. Any product that may be evaluated in this article, or claim that may be made by its manufacturer, is not guaranteed or endorsed by the publisher.
