# Supplementary material for: Arrhythmias in Patients With Valvular Heart Disease: Gaps in Knowledge and the Way Forward
Source: Front Cardiovasc Med. 2022 Feb 15;9:792559. doi: 10.3389/fcvm.2022.792559 (PMC8885812; doi:10.3389/fcvm.2022.792559)
Supplement: Supplementary file 1 [file Table_1.docx]

**Supplementary Table 1. Warranted clinical trials of detection and treatment of valvular heart disease – associated arrhythmias**

| **Trials of arrhythmia detection** | Prevalence and patterns of AF in patients with moderate/severe VHD using loop recorder  Prevalence, character and site of origin of ventricular arrhythmia in moderate/severe aortic stenosis using 12 lead Holter and treadmill test and cardiac MRI  Stratification of the risk of SCD in arrhythmogenic mitral valve prolapse using treadmill test, programmed ventricular stimulation, and loop recorder with remote monitoring  Incidence of AF after valvular replacement/repair in VHD subtypes using loop recorder  Burden of PVCs in mild MR  Burden and character of VAs following valvular surgery using loop recorders/12 lead Holters and treadmill test  Impact of early VTs following valvular surgery on the risk of recurrences and mortality |
| --- | --- |
| **Trials of arrhythmia treatment** | Catheter ablation vs medical treatment for AF in moderate VHD  Timing of catheter ablation vs medical treatment for AF in severe high-risk VHD  Timing of catheter ablation for AF in severe intermediate-risk VHD  Pulmonary veins isolation only or with additional lines/ Extra-venous ectopies for patients with persistent AF and moderate VHD  Impact of preoperative AF burden/patterns and the left atrial volume on the results of adjunctive surgical ablation of AF using preoperative monitoring (Holter, loop recorder) and echocardiography  Results of hybrid AF ablation in patients undergoing valvular surgery  Antiarrhythmic therapy for PVCs in cases of progressive LV enlargement in patients with mild organic MR  Beta-blockers for the prevention of SCD in the setting of organic VHD |

AF, atrial fibrillation; CA, catheter ablation; SCD, sudden cardiac death; VHD, valvular heart disease; VT, ventricular tachycardia
