# Supplementary material for: Arrhythmias in Patients With Valvular Heart Disease: Gaps in Knowledge and the Way Forward
Source: Front Cardiovasc Med. 2022 Feb 15;9:792559. doi: 10.3389/fcvm.2022.792559 (PMC8885812; doi:10.3389/fcvm.2022.792559)
Supplement: Supplementary file 2 [file Table_2.docx]

**Supplementary Table 2. Overview of relevant studies that have reported results of catheter ablation of ventricular arrhythmias for patients with concomitant valvular heart disease.**

| **Reference** | **Author** | **Sample Size** | **Age (y)** | **Female (%)** | **Valvular abnormality (%)** | **Type of arrhythmia** | **Common ablation site** | **Acute success**  **(%)** | **Mean Follow-up (months)** | **Follow-up**  **Results** |
| --- | --- | --- | --- | --- | --- | --- | --- | --- | --- | --- |
|  |  |  |  |  |  |  |  |  |  |  |
| 91 | Syed et al | 14 | 33.8 | 93 | Bileaflet MVP with  ≤ moderate MR | NSVT or sustained VT (57%)  Prior cardiac arrest ICD shocks for PVC-triggered VF (43%) | LV papillary muscle/fascicular  system (93%)  Both papillary muscles (55%)  Purkinje origin (79%) | 86 | 25 | Significant reduction in VT burden and appropriate ICD shocks |
|  |  |  |  |  |  |  |  |  |  |  |
| 88 | Lee et al | 9 | 58.0 | 78 | Bileaflet MVP (89%)  Mild MR (67%) | NSVT (56%) | Postero-medial papillary muscle (48%)  Both LV papillary muscles (26%) | 60 | 41 | VA recurrences for 25%* |
|  |  |  |  |  |  |  |  |  |  |  |
| 87 | Bumgarner  et al | 30 | 54.3 | 53 | Bileaflet MVP (52%)  Posterior MVP (36%)  ≥ 2 MR (72%) | PVC (44%)  Sustained VT (39%) | Papillary muscle (27%)  MV annulus (15%) | 67 | 30 | VA recurrences for 26% |
|  |  |  |  |  |  |  |  |  |  |  |
| 46 | Enriquez et al | 25 | 54.7 | 64 | Bileaflet MVP (72%)  Mild to moderate MR (76%) | PVC and NSVT (56%) | Postero-medial papillary muscle (56%)  Antero-lateral papillary muscle (32%) | 76 | 31 | 20% → 6%  decrease in PVC burden |
|  |  |  |  |  |  |  |  |  |  |  |
| 106 | Eckart et al | 14 | 53.0 | 0 | Post AVR | Sustained VT | Antero-septal scar related reentry (36%)  Lateral LV scar (27%) | 100 | 25 | VA recurrence for 14% |
| 118 | Liang et al | 29 | 67.9 | 13 | Post AVR (55% bioprosthetic) | Sustained VT | Periaortic scar reentry (79%) | 100 | - | VT recurrence for 27% |
|  |  |  |  |  |  |  |  |  |  |  |

* VA recurrence rates reported after single CA procedure

AVR, aortic-valve replacement; ICD, implantable cardioverter-defibrillator; LV, left ventricle; MR, mitral regurgitation, MV, mitral valve; MVP, mitral-valve prolapse; NSVT, non-sustained ventricular tachycardia; PVC, premature ventricular complex; VA, ventricular arrhythmia; VT, ventricular tachycardia.
